# Supplementary material for: PolyCRACKER, a robust method for the unsupervised partitioning of polyploid subgenomes by signatures of repetitive DNA evolution
Source: BMC Genomics. 2019 Jul 12;20:580. doi: 10.1186/s12864-019-5828-5 (PMC6626429; doi:10.1186/s12864-019-5828-5)
Supplement: Supplementary file 1 — Supplementary data. This files contains supplementary data about polyCRACKER performance on simulated datasets comprised of mixtures of subsequences from multiple closely-related species and highly differential repeat subclasses between N. Tabacum subgenomes. (DOCX 380 kb) [file 12864_2019_5828_MOESM1_ESM.docx]

**Supplementary data**

**polyCRACKER performance on simulated datasets comprised of mixtures of subsequences from multiple closely-related species**

Three simulated datasets created from reference fungal or algal genomes were used to develop and test polyCRACKER. The genomes were chosen to cover a range of repeat contents and genomes sizes. The simulated datasets were created by fragmenting and pooling the reference genomes to simulate the contigs expected from anallopolyploid genome. PolyCRACKER was then optimized to correctly assign the sequence fragments to their genome of origin.

The first simulated dataset contained genomes from two closely related (mean nucleotide identity of CDS, 77%) Basidiomycte fungi, Ustilago hordei [17] and U. maydis, whose small (~20Mb) genomes contain only 7.8% and 2% repetitive DNA, respectively [17, 18]. Genomes of the two species were broken into 50 kb fragments, labeled by species (for subsequent calculation of accuracy), and then combined into a single FASTA file. Without any other input, polyCRACKER assigned sequences to the correct species with perfect precision and 99% recall (Supplemental Table 1).

The second simulated dataset contained four closely related (pairwise nucleotide identity of CDS ranged from 63-70%) Ascomycete fungi: *Aspergillus* *glacus* (30 Mb, 3% repetitive), *A. aculeatus* (35 Mb, 4.5% repetitive), *A. versicolor* (33 Mb, 1.8% repetitive), and *A. wentii* (34 Mb, 1.8% repetitive) [19]. The four genomes were broken into 100 kb fragments and pooled as for the first dataset. PolyCRACKER assigned the sequences to the correct species with 95 percent precision and 90 percent recall (Supplemental Table 1).

Lastly, we tested our method on a simulated dataset containing two single-cell green algae: *Chlamydomonas reinhardtii* [20] (16% repetitive DNA) and *Coccomyxa subellipsoidea*] (1.5% repetitive DNA). The simulated dataset was created exactly as for the first dataset. PolyCRACKER assigned the sequences to the correct species with 93 percent precision and 92 percent recall (Supplemental Table 1).

The dimensionality reduction and clustering techniques used to establish the initial species bins are depicted in Supplemental Table 2. The amount of repetitive k-mers in each of the genome fragments of the two algae lines’ fragmented assemblies is shown in Supplemental Figure 1. It is fairly low as compared to *N. Tabacum’s* distribution of its repetitive content, after scaling for fragment length (Figure 5).

| **Supplemental Table 1** Unsupervised separation of simple mixtures of fungal and algal genomes using PolyCRACKER’s differential k-mer analysis. | | | | | | |
| --- | --- | --- | --- | --- | --- | --- |
| Species | Fragment size | k-mer length | Precision | Recall | F1 Score | Accuracy |
| *Aspergillus aculeatus, A. glaucus, A. versicolor,* and *A. wentii* | 100 kb | 11 | 0.95 | 0.9 | 0.92 | 0.9 |
| *Ustilago hordei,* and *Ustilago maydis* | 50 kb | 15,30,45* | 1 | 0.99 | 0.99 | 0.99 |
| *Chlamydomonas subellipsoidea,* and *Chlamydomonas reinhardtii* | 50 kb | 26 | 0.93 | 0.92 | 0.92 | 0.92 |

* Three separate k-mer lengths were used in this analysis because a greater repetitive k-mer signal was found at different k-mer lengths over various subsets of subsequences.

| **Supplemental Table 2** Dimensionality reduction and clustering techniques used in polyCRACKER for the simulated metagenomes. | | |
| --- | --- | --- |
| Species | Dimensionality reduction technique | Clustering technique |
| *A. aculeatus, A. glaucus, A. versicolor,* and *A. wentii* | TSNE | Bayesian Gaussian Mixture Models |
| *U. hordei* and *U.maydis* | KPCA with linear kernel | Bayesian Gaussian Mixture Models |
| *C. subellipsoidea* and *C. reinhardtii* | TSNE | Spectral Clustering |


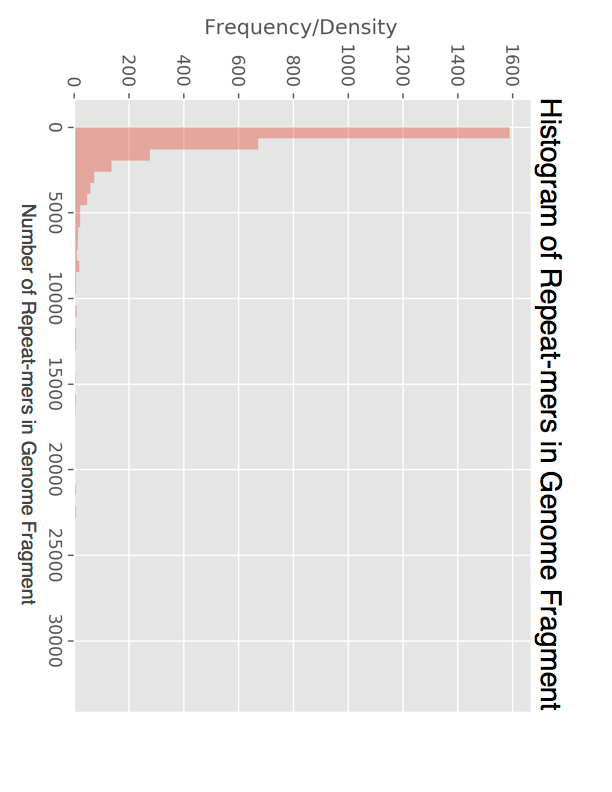


**Supplemental Fig. 1.** Distribution of the number of repeat-mers in the genome fragments of the two single-cell green algae assemblies. These numbers were derived from counting the total number of repetitive k-mers in each genome fragment after breaking the assembly into fragments of a fixed size.

**Highly differential repeat subclasses between *N. Tabacum* subgenomes**

Two hundred informative differential repeat consensus sequences were chosen for each subgenome (S and T) of the unanchored scaffold set via the same process used to identify highly informative differential repeats, and analyzed for their subclasses and the subgenome specificity of these subclasses. All repeats including the top differential repeats were broken down into their class or subclass and the categorical distribution of all consensus repeats were used as the null distribution. The categorical distribution of the top differential repeats were compared to the null distribution, and expected number of top differential repeats were found for each subclass. The resulting distributions with the top four chi-squared values are displayed in Supplemental Table 3. The top subclass, Unknown, with a highest chi-squared value of 780.74, was found, and all top differential Unknown consensus sequences were broken down into their subgenomes and a phylogenetic tree was constructed (Supplemental Figure 2). The same analysis was done for top subclass three, LTR/Gypsy, but not subclass two, since Simple Repeats are very small and aligning them yields little to no new information about their clade structure. However, it should be noted that Simple Repeats were entirely T subgenome enriched.

| **Supplemental Table 3** PolyCRACKER repeat analysis. | | | | |
| --- | --- | --- | --- | --- |
|  | LTR Copia | LTR Gypsy | Simple repeat | Unknown repeat |
| Number of consensus sequences | 151 | 527 | 10,694 | 1,414 |
| Number included in the 400 most informative repeats | 5 | 70 | 90 | 229 |
| Expected number among 400 most informative repeats | 4.68 | 16.35 | 331.93 | 43.88 |
| Chi-Squared Value | 0.02 | 175.9 | 176.3 | 780.7 |
| Top 400: Number of S subgenome to T subgenome consensus repeats | 1:4 | 43:27 | 0:90 | 152:77 |


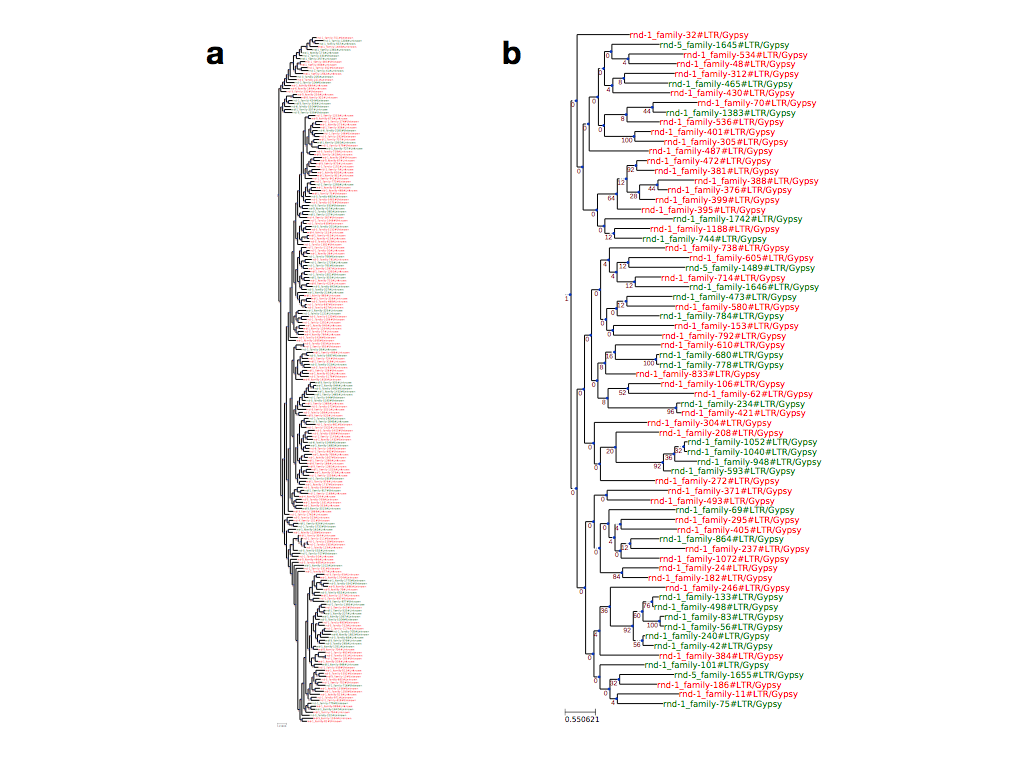


**Supplemental Fig. 2.** Specific repeat classes contribute to the differentiation of the *N. tabacum* subgenomes. IQTree GTR trees for top subclasses of *N. tabacum* genome assembly. The top three differential classes are **(a)** transposons with unknown class enriched in the S subgenome (red) **(b)** tranLTR/Gypsy (S subgenome enriched). Phylogenetic trees were constructed using maximum likelihood General Time Reversible (GTR) model of evolution, performed with 25 bootstrap iterations.
